# Supplementary material for: Forget binning and get SMART: Getting more out of the time-course of response data
Source: Atten Percept Psychophys. 2019 Jun 18;81(8):2956–67. doi: 10.3758/s13414-019-01788-3 (PMC6856044; doi:10.3758/s13414-019-01788-3)
Supplement: Supplementary file 1 — (DOCX 423 kb) [file 13414_2019_1788_MOESM1_ESM.docx]

Forget binning and get SMART: Getting more out of the time-course of response data

Jonathan van Leeuwen^1*^ [jvanleeuwen.work@gmail.com](mailto:jvanleeuwen.work@gmail.com),
Jeroen B. J Smeets^2^ and
Artem V. Belopolsky^1^

^1^Department of Experimental and Applied Psychology
Vrije Universiteit
Amsterdam, The Netherlands

^2^Department of Human Movement Sciences
Vrije Universiteit
Amsterdam, The Netherlands

Abstract

Many experiments aim to investigate the time-course of cognitive processes while measuring a single response per trial. A common first step in the analysis of such data is to divide them into a limited number of bins. As we demonstrate here, the way one chooses these bins can considerably influence the resulting time-course. As a solution to this problem, we here present the smoothing method for analysis of response time-course (SMART)—a complete package for reconstructing the time-course from one-sample-per-trial data and performing statistical analysis. After smoothing the data, the SMART weights the data based on the effective number of data points per participant. A cluster-based permutation test then determines at which moments the responses differ from a baseline or between two conditions. We show here that, in contrast to contemporary binning methods, the chosen temporal resolution has a negligible effect on the SMART reconstructed time-course. To facilitate its use, the SMART method, accompanied by a tutorial, is available as an open-source package.

Keywords

Statistics
Reaction time methods
Perception and action

Many experiments aim to investigate the time-course of cognitive processes by measuring the resulting performance as a function of time. For example, researchers have been interested in whether fast decisions are less accurate than slow decisions (Heitz, [2014](#B6); Henmon, [1911](#B7)). To answer this question, one could separate fast responses from slow responses, and then examine whether they differ in accuracy (Henmon, [1911](#B7)). However, such a crude approach allows only for a sneak-peek into the time-course of the decision process, while the real time-course of decision-making would remain a mystery. Fully understanding the dynamics of a cognitive process requires reconstructing its time-course from the available data. The challenge lies in the fact that in most behavioral experiments, the time-course of a cognitive process is sampled on a one-sample-per-trial basis. In the example above, each trial would contain a single response measure (e.g., correct or incorrect) sampled at a certain (response) time that will be different for every trial. In the present paper, we present a novel method for visualizing and analyzing such data as a time-series, similar to how EEG data are typically visualized and analyzed.

For more than 100 years, the standard method for creating a time-course of one-sample-per-trial data has been *binning* (Henmon, [1911](#B7); Ratcliff, [1979](#B12); Vincent, [1912](#B16), as cited in Ratcliff, [1979](#B12)). This method prescribes dividing the time variable into several bins. For each trial, the data from the response variable are then allocated to the respective bins. The data per bin are then collapsed (typically, an arithmetic mean is taken) to produce a single data point per bin. The resulting values yield a time-course per condition for each participant, which can be collapsed across participants to construct the time-course of interest.

There are two main approaches to data binning in the literature. The first is named “Vincentizing,” after Vincent ([1912](#B16)), and probably the most popular approach (but see Rouder & Speckman, [2004](#B13), for a critical evaluation). In Vincentizing, the bins are created by dividing the time variable into several contiguous intervals with an equal number of trials for each participant, so that the performance data can be analyzed across participants with maximal power. The second method of binning involves creating several contiguous time intervals that are the same for each participant (Henmon, [1911](#B7)).

Both binning methods suffer from a number of problems, ranging from signal distortion and reduction in temporal resolution to complications for statistical analysis. Below, we provide a detailed analysis of the problems of using these two methods. The goal of this paper is to introduce the smoothing method for analysis of response time-course (SMART) method—a new data analysis method that provides a complete package for reconstructing the time-course from one-sample-per-trial data and performing statistical analysis. We demonstrate the capabilities and advantages of our method by contrasting it with two binning methods using two existing experimental datasets.

1. Binning methods

When applying the Vincentizing method, it is important to be aware of the crucial assumption that the time-course of the dependent variable (e.g. the accuracy) of all participants are distributed over the same phase of the cognitive process (see Fig. 1, left column). When averaging such a time-course across participants, the temporal pattern of the cognitive process can be accurately reconstructed using Vincentizing (see Fig. 1, left column, third row). However, this assumption is violated if the dependent variable is time-locked to an external event (e.g., a neural response to visual or auditory cue), but the response times (and thus the resulting performance) vary between participants (see Fig. 1, right column). If the assumption of a participant-specific data distribution for the dependent variable is violated and participants have very different ranges of response time, averaging using Vincentizing can dramatically distort the reconstruction of the time-course (see Fig. 1, right column). As the bins are determined by each participant’s distribution of response times, the borders of the bins will be different for each participant. Therefore, a signal that has a sigmoid shape can appear linear and show only a fraction of the original variation after reconstruction by Vincentizing. To make such variability in the timing of a bin across participants visible for the reader, horizontal error bars should also be included in the time-course plot (see Fig. 3a , Experiment 1, Boon, Zeni, Theeuwes, & Belopolsky, [2018](#B1), for an example), but this is often forgotten (Godijn & Theeuwes, [2002](#B5); Silvis, Belopolsky, Murris, & Donk, [2015](#B14)).

The “hard-limit binning” method assumes that every data point from every participant represents a sample from the same general distribution—that is, an event-locked dependent variable (see Fig. 1, right column). One consequence of this method is that if participants have widely different response distributions, participants do not contribute equally to each bin. In extreme cases, several bins may lack data from some participants, while other bins are composed of data from all participants. This may also introduce distortions to the average time-course when an arithmetic mean of each bin is taken. Most importantly, however, is that if the participants do not contribute equally to each individual bin, performing adequate statistics for each bin becomes questionable.

Fig. 1 A simulated dependent variable as a function of time and the reconstruction of the simulated time-course by binning data. The left column shows a simulated dependent variable with a participant-specific signal and responses distributed over the relevant part of the process for each of the five participants. The right column shows a simulated event-locked signal for five participants, but their response-time distributions differ. The panels in both columns, from top to bottom, show raw sample data, per participant Vincentized bins, averaged Vincentized bins, per participant hard-limit bins, and averaged hard-limit bins. Averaged across participants, the time-course of the dependent variable is reconstructed well by Vincentizing for the participant-specific timing, and by hard-limit bins for the event-locked timing. (Color figure online)

1. The SMART method

The response times of individual responses can be measured with millisecond precision. Thus, the raw data has high temporal precision. Using either of the two binning methods severely reduces the temporal resolution as the number of bins limits it. Often, the number of bins is chosen arbitrarily. Furthermore, the chosen number of bins may complicate the statistical analysis of the time-course. Notably, the power of the statistical analysis will inevitably decrease with increasing number of bins. Therefore, while reducing the temporal resolution, a low number of bins is often chosen to keep sufficient statistical power. To alleviate the aforementioned problems with binning, a moving window averaging method was introduced (Maij, Brenner, & Smeets, [2009](#B9)). With this method, data for each participant is smoothed using a moving Gaussian kernel as a function of time. If a narrow kernel is used, the temporal resolution of a dependent variable reconstructed by this method is considerably better than the temporal resolution obtained by binning. The method is flexible, as the size and type of kernel can be adjusted depending on the type and quantity of data (Maij, Brenner, Li, Cornelissen, & Smeets, [2010](#B8); Maij et al., [2009](#B9)). To prevent effects of outliers, Maij and colleagues excluded clusters of samples in the smoothed time-course if there were not enough data. For instance, Maij et al. ([2009](#B9)) excluded samples if there were less than five data points within two times the standard deviation away from the peak of the Gaussian. This approach can make it difficult to average the time-course across participants if there is missing data.

To answer the question “At what moment the dependent variable differs from a baseline?”, one could perform a *t* test for each time point in the smoothed data with an appropriate correction for multiple comparisons. However, a Bonferroni correction is not appropriate here, as the dependent variable at adjacent time points are not independent of each other. One way to solve both the multiple comparisons problem and the problem of dependence between data points, is to use clusters of several temporally adjacent time points that show a significant difference in the dependent variable instead of using individual time points. If the strength of the effect, summed over the cluster, is larger than a certain minimum strength, the effect is considered to be significant. This procedure is used in the analysis of EEG data (Maris & Oostenveld, [2007](#B11)). When analyzing EEG data, choosing a minimum cluster strength to correct for multiple comparisons is usually done by using cluster-based permutation testing (Fahrenfort, van Leeuwen, Olivers, & Hogendoorn, [2017](#B3); Maris & Oostenveld, [2007](#B11)). However, application of the cluster-based permutation testing to smoothed one-sample-per-trial data is not trivial, since unlike EEG data, the data do not contain a time-series per trial, and thus many time-series per participant, but rather consists of a single smoothed time-series per participant.

Below we introduce the SMART method. It consists of three major parts: (1) temporal smoothing, (2) weighted statistics that takes into consideration the contribution by each participant, and (3) permutation testing. Two different versions of the statistics and permutation tests are presented: (a) for determining when time points show a significant difference in the dependent variable from a baseline (weighted one-sample t-test) and (b) for determining when time points show a significant difference in the dependent variable between two conditions (weighted paired-sample *t* test).

1. Part 1: Temporal smoothing and averaging across participants

The first part of the analysis is the temporal smoothing of one-sample-per-trial data (see Fig. 2). Since the dependent variable is sampled once per trial, each trial returns a single data point consisting an independent measure such as (reaction) time and a dependent variable such as performance (fraction correct, saccade curvature, etc.). The pairs of *time* and *performance* data points aggregated across all trials per participant serve as input for the temporal smoothing procedure. By repeating the first and second step (see detailed explanation below) for each participant, the time-series per participant is constructed. In the third step, a weighted averaging across participants result in a group-average time-series. Below, we present the procedure for temporal smoothing (see Fig. 2).

The first step is to organize the data from all trials (*N*) per participant (*i*) in pairs, linking the performance $P_{i}\left( n \right)$on trial (*n*) with the time $\tau_{i}\left( n \right)$ on that trial: {$P_{i}\left( n \right)$, $\tau_{i}\left( n \right)$} (see Fig. 2a).

The second step is to reconstruct the estimated time-series for each participant by convolving the performances$P_{i}\left( n \right)$ with a Gaussian kernel of a width *σ* (see Fig. 2a):

$P_{i}\left(t\right)=\sum_{n=1}^{N} \left(\frac{P_{i}\left(n\right) e^{-\frac{\left(\tau_{i}\left(n\right)-t\right)^{2}}{2\sigma^{2}}}}{w_{i}\left(t\right)}\right).$ (1)*

In Eq. 1, the estimated performance at a specific time point $P_{i}\left( t \right)$ is multiplied by the kernel density estimate depending on the time-difference (determined by a Gaussian; see Fig. 2a). In order to scale the output value to the same scale as the original data, we divide this by $w_{i}\left( t \right)$, the sum of the kernel density estimates with which the trials contribute at time (*t*) for participant (*i*) (see Fig. 2a, gray insert):

$w_{i}\left(t\right)=\sum_{n=1}^{N} e^{-\frac{\left(\tau_{i}\left(n\right)-t\right)^{2}}{2\sigma^{2}}}.$ (2)

The third step is to average across participants. In this averaging, we ensure that participants with more data around certain time points contribute more to the group average than participants with fewer data around the same time points. This is achieved by multiplying each participant’s estimated performance $P_{i}\left( t \right)$ with the corresponding normalized weight $\boldsymbol{W}_{i}\left( t \right)$ (see Fig. 2a, gray insert):

$\overline{P_{W}}\left(t\right)=\sum_{i=1}^{NP} W_{i}\left(t\right) P_{i}\left(t\right).$ (3)

With $\boldsymbol{W}_{i}\left( t \right)$ being the normalized weight that ensures that participants with more data around certain time points contribute more to the group average than participants with fewer data around the same time points:

$W_{i}\left(t\right)= w_{i}\left(t\right)/\sum_{i=1}^{NP} w_{i}\left(t\right).$ (4)

Fig. 2 Schematic illustration of the SMART smoothing procedure. **a** Smoothing for data from one hypothetical participant with nine trials using Eq. 1. Gray insert: Calculating the weight of each smoothed time point for a participant, given by Eq. 2. $w_{i}\left( t \right)$ reflects the sum of kernel density estimates under each Gaussian curve at the sample time point *t*. **b** Constructing a weighted average time-course. The data is weighted across participants for each time point. Using Eqs. 3 and 4. The stars reflect the smoothed samples along the time axis. The black stars with connecting black lines equal time points which differ significantly from baseline, given Eq. 5 for testing against a baseline and Eqs. 6 to 8 for paired-sample testing. (Color figure online)

1. Part 2: Weighted statistics

Since the data are smoothed to a weighted time-series, the statistics that we apply for each time point necessarily needs to be weighted statistics (see Fig. 2c). Estimated confidence intervals for one-sample and paired-sample tests are determined by multiplying the corresponding standard errors of the weighted mean by the *t* value corresponding to the desired Type I error (alpha) in the *t* distribution with *NP* − 1 degrees of freedom. The calculations required for weighted statistics differ between one-sample testing (against baseline) and paired-sample testing and are described separately below.

Note that there is no analytical solution for the standard error of the weighted mean. While several approximations exist, here we use the ratio variance approximation, as described in Gatz and Smith ([1995](#B4)), which has been demonstrated to be statistically indistinguishable from the standard error estimates obtained through bootstrapping. We have also verified that the approximation applies to the current data by performing bootstrapping and comparing the estimates (see Supplementary Materials, Fig. [S1](#F8)). The main advantages of using the approximation is that it requires less effort and less computation time.

- 1. One-sample testing

$SEM_{W}\left(t\right)=\sqrt{\frac{NP}{NP-1}\sum_{i=1}^{NP} \left(W_{i}\left(t\right)\left(P_{i}\left(t\right)-\overline{P_{W}}\left(t\right)\right)\right)^{2}}.$ (5)

For one-sample *t* test, the estimated standard error of the weighted mean for each time point${SEM}_{W}\left( t \right)$, is approximated by:

- 1. Paired-sample testing

For paired-sample testing, the estimated difference of the standard error of the weighted mean ${\Delta SEM}_{\boldsymbol{W}}\left( t \right)$ is approximated by:

$\Delta SEM_{W}\left(t\right)=\sqrt{\frac{NP}{NP-1}\sum_{i=1}^{NP} \left(\left(W_{i}^{\left[A\right]}\left(t\right)W_{i}^{\left[B\right]}\left(t\right)\right)\left(\Delta P_{i}\left(t\right)-\overline{\Delta P_{W}}\left(t\right)\right)^{2}\right)},$ (6)

where ${\boldsymbol{W}_{i}}^{\left[ A \right]}(t)$ is the normalized weight for condition A and ${\boldsymbol{W}_{i}}^{\left[ B \right]}(t)$ is the normalized weight for Condition B at time $(t)$. ${\Delta P}_{i}\left( t \right)$ is the difference in the nonweighted average performance estimate between Condition A and Condition B for each participant; it is given by:

$\Delta P_{i}\left(t\right)= P_{i}^{\left[A\right]}\left(t\right)- P_{i}^{\left[B\right]}\left(t\right),$ (7)

and where $\bar{{\Delta P}_{W}}\left( t \right)$ is the average weighted performance difference between Condition A and Condition B:

$\overline{\Delta P_{W}}\left(t\right)=\sum_{i=1}^{NP} \left(W_{i}^{\left[A\right]}\left(t\right)P_{i}^{\left[A\right]}\left(t\right)- W_{i}^{\left[B\right]}\left(t\right)P_{i}^{\left[B\right]}\left(t\right)\right).$ (8)

1. Part 3: Cluster-based permutation testing

When analyzing the time-course of a cognitive process, an important question is when a dependent variable differs from a baseline or when the dependent variable differs between two conditions. If the data are binned, traditional methods such as ANOVA can be used to answer these questions by determining whether there is a main effect of Bin or a Condition × Bin interaction. However, to investigate an onset or an offset of a certain event, a post hoc analysis per bin is often performed. Such a post hoc analysis requires some form of correction for multiple comparisons, such as the Bonferroni correction. For an analysis with only a few bins, the temporal resolution is poor; when a large number of bins is used, Bonferroni correction will become too conservative, and greater statistical power will be required to reject the null hypothesis. Obviously, the latter objection also holds when an ANOVA is applied to the individual time points of the smoothed time-course. Since the dependent variable is not independent of its value at a neighboring time point, clusters of contiguous time points that show a significant difference in the dependent variable will emerge. Therefore, instead of determining whether differences at each individual time point are significant, one needs to determine whether the difference is significant for a given cluster, several temporally adjacent time points that show a significant difference in the dependent variable. To solve this problem, cluster-based permutation testing has been developed, a technique that is a widely used method in neuroimaging (Bullmore et al., [1999](#B2); Fahrenfort et al., [2017](#B3); Maris & Oostenveld, [2007](#B11)).

Permutation analysis involves building the distribution of the test statistic under the null hypothesis by calculating the values of the test statistic for all rearrangements of labels on the observed data points. In electrophysiology the trial labels are shuffled between conditions, thereby averaging out any effect of condition. For each permutation, the clusters are computed and the strength of the largest cluster (e.g., the test statistic) is noted. Repeating the permutation process many times allows building the distribution of cluster strengths under the null hypothesis. Comparing the cluster strength of the nonpermuted data to the cluster strength distribution in the permuted data it is possible to determine the significance level—for example, the test statistic corresponding to the 95th percentile (or a *p* value of .05; see Fig. 3). Any cluster in the nonpermuted data with a cluster strength higher than the 95th percentile is a significant cluster.

Applying the cluster-based permutation to the EEG data is straightforward because each trial contains an entire time-series. In contrast, applying the cluster-based permutation to one-sample-per-trial data is not trivial, as all the trials are needed to create a single time-series per participant. Therefore, after shuffling the labels for each trial, the entire smoothing procedure needs to be repeated for each permutation. Additionally, shuffling trials need to be performed differently, depending on whether one is testing significance between two conditions (paired-sample test) or against a baseline (one-sample test). These two cluster-based permutation methods are discussed separately below.

- 1. Permutation testing: Between conditions and against baseline

The SMART procedure for cluster-based permutation testing between two conditions is divided into six steps. The goal of the permutation testing between conditions is to determine the probability of observing the test statistic under the null hypothesis. In this case, *the first step* is, therefore, to combine the data from both conditions within a participant (see Fig. 3b).

When using the SMART procedure for testing against baseline, there is only a Condition A, no second condition. Therefore, in this case, *the first step* involves creating a second “baseline” condition (Condition B). The time values (and thus the number of trials) for Condition B are the same as the time values in Condition A. All the values for the dependent variable are set to the baseline value you would like to compare the data against. One possible concern is that using a baseline without variance might influence the results of the cluster-based permutation testing by underestimating the cluster size required to achieve statistical significance. However, adding noise to the baseline in our cluster-based permutation procedure does not result in different cluster distributions (see Fig. [S2](#F8) and Table [S1](#F8) in the Supplementary Materials). Thus, by creating a baseline signal, we have two conditions: Condition A with the measured data and condition with an equal number of data points with all the values set to the baseline value. Therefore, we can combine the conditions in the same way as for testing between conditions, and *all subsequent steps* are identical for between-conditions testing and against baseline testing.

Because the question of interest is whether the dependent variable differs between the two conditions, *the second step* is to permute the data by shuffling the trials in the combined data.

*The third step* is to create two new permuted datasets, one for each condition. This is done by extracting the same number of trials from the combined and permuted data for each condition as there were trials in that condition (e.g., if Condition A had 30 trials and Condition B had 42 trials, then one permuted condition would have 30 trials and the other permuted condition would have 42 trials; see Fig. 3b). After extracting the permuted conditions, each permuted condition is run through the Gaussian smoothing procedure (see Fig. 2a). This step is repeated for *N* permutations for each participant. Each permutation results in a new smoothed time-course and corresponding weights for each participant.

*The fourth step* is to perform the weighted group-level statistic (weighted paired-sampled *t* test) for each time point in each permutation. This is the same group-level test as was performed for the nonpermuted smoothed data. This results in a *p* value and a *t* value for each sample for each of the permuted time-series (see Fig. 3c).

*The fifth step* is to select the strongest cluster for each permutation. If there are no clusters in a given permutation, the largest *t* value in that permutation is used as cluster strength, and thus the total number of clusters in the final cluster distribution is equal to the number of permutations (see Fig. 3c).

*The sixth step* is to determine the 95th percentile of the obtained distribution of cluster strengths. Any cluster in the nonpermuted data whose strength is equal to or is larger than the 95th percentile of the permuted distribution constitutes a significant cluster (see Fig. 3c). The nonpermuted cluster’s *p* value is therefore given by 1 -the percentile of the nonpermuted cluster in the permuted distribution.

Fig. 3 The SMART analysis procedure. **a** Procedure overview. **b** Building the permutation distribution. **c** Performing statistical analysis and determining significance threshold. (Color figure online)

1. Testing the SMART method on experimental datasets

To demonstrate the differences in the estimated time-course between the two methods of binning data and the SMART method described above, the data from two different studies were analyzed (see Figs. 4 and 5). The dataset from Silvis et al. ([2015](#B14)) was used for testing performance between conditions (see Fig. 3) and the dataset from van Leeuwen and Belopolsky ([2018](#B15)) was used for testing performance against baseline. Possible theoretical implications of the results of this reanalysis for the individual studies will not be discussed, as this is beyond the scope of the current manuscript. A good method is robust under variations of parameter that can be chosen freely by the experimenter. For all three methods, the experimenter can choose the temporal resolution freely. Therefore, we ran the analysis for five different values for the parameter that influences the temporal resolution: five number of bins (3, 4, 5, 6, 7) for Vincentizing and hard-limit bins and five values of the σ (50 ms, 40 ms, 30 ms, 20 ms, and 10 ms) for the SMART method. By running the analysis with different temporal resolutions and comparing the temporal estimates for each temporal resolution, we can estimate which one of the methods is least sensitive to this parameter change. When using the Vincentizing method, the data are split into equally sized bins (Vincent, [1912](#B16)). For the hard-limit bins, we split the period of interest into equally sized bins. For both binning methods, a Bonferroni-corrected *t* test was performed to test whether performance differed in a bin.

To compare the temporal estimates as a function of temporal resolution, we estimated for each temporal resolution the time at which performance changed. We subsequently calculated the mean and the standard deviation of these five estimates. A low standard deviation of the estimates would indicate that the temporal resolution only has a small influence on the temporal estimate and would suggest that the arbitrary choice of temporal resolution does not meaningfully influence the temporal estimate extracted from the new time-series. The temporal resolution for the SMART method is directly determined by the value for σ, whereas the temporal resolution of binning methods is determined by the width of the bins, which is either more or less arbitrarily chosen (hard-limit) or emergent (Vincentizing) given the number of bins and the temporal distribution of the data. Thus, the temporal resolution for a certain number of bins does not perfectly match with a single value for σ. For the SMART method, we ran 1,000 permutations for each value of σ for each dataset.

- 1. Dataset 1—Fraction correct

The first dataset comes from an experiment that examined the interplay between feature-based priming and oculomotor capture (Silvis et al., [2015](#B14)). They presented participants with a colored square that participants had to memorize. Subsequently, two bars with different orientations were presented. Participants had to make an eye movement to the bar with a specific orientation. Either the target bar or the distractor bar matched the color that was memorized. A trial was classified as correct of the participant made an eye movement to the bar with the correct orientation and incorrect if they made an eye movement to the bar with the incorrect orientation. Of interest was the response time for which the difference in performance between the target match and distractor match trials disappeared. The data are analyzed using the SMART procedure for determining differences between conditions (see Fig. 3). For the hard-binning method, we set the lower and upper limits for the hard-limit binning to 100 ms and 500 ms, respectively. Note that the dependent measure is binary (correct/incorrect) and that there was a third condition in their experiment which is omitted in our analysis for the sake of simplicity. The total number of trials used in the analysis was 4,620.

- 1. Data set 2—Saccade curvature

The second dataset comes from an experiment that examined saccade curvature in a double-step saccade paradigm. We used the data from the condition in which the distractor was displaced during a saccade (merged data from Experiments 1 and 2, as plotted by the dark red curve in Fig. 6 of van Leeuwen & Belopolsky, [2018](#B15)). Of interest was the intersaccadic interval for which the saccade curvature of the second saccade changed sign. This dataset was analyzed using the SMART procedure for determining differences from baseline. For the hard-binning methods, we set the lower and upper limits for the hard-limit binning to 100 ms and 300 ms, respectively. Note that saccade curvature is a continuous measure and the baseline is zero saccade curvature. The total number of trials used in this analysis was 23,968.

1. Results
   1. Dataset 1—Fraction correct

Dataset 1 was used to demonstrate the analysis of a within-participants experiment with permutation testing between conditions. In the original results, the target match condition and the distractor match condition show the largest difference at early saccade latencies and then slowly converge at later saccadic latencies (Silvis et al., [2015](#B14)). The goal of the present analysis is to determine at what saccadic latency the two conditions no longer differ from each other.

Fig. 4 Results for Dataset 1. Columns 1–3: The proportion correct saccades as a function of saccade latency when using Vincentizing, hard-limit bins, and SMART, respectively. Cyan indicates the performance of the distractor match condition and dark red indicates the performance of the target match condition. Vertical error bars and shaded areas indicate the 95% confidence intervals. Horizontal error bars indicate the standard deviation of the mean time for each bin across participants. The number in the upper right corner indicates the number of bins or the value for σ. The asterisks in Columns 1 and 2 indicate bins that differ significantly between conditions at *p* < .05, Bonferroni corrected. In Column 3, the black lines indicate time points at which the two conditions differ significantly from zero, and asterisks indicating which clusters are statistically significant. The dark-gray shaded area is the estimated number of trials per millisecond (right axis), for the target match condition. The light-gray shaded area (completely occluded) is the estimated number of trials per millisecond (right axis) for the distractor match condition. Estimated with the same kernel size as the one used for the SMART procedure. Column 4: The permutation distribution between conditions. The blue histogram shows (on a logarithmic scale) the frequency of the sum of *t* values of clusters in the permuted time-series. The vertical red line indicates the 95th percentile for the permuted time-series. The vertical black lines indicate the sum of cluster *t* values in the nonpermuted time-series. (Color figure online)

As expected, all three methods replicate the findings by Silvis et al. ([2015](#B14)): The target match conditions shows continuously high saccade accuracy for all saccade latencies, while the distractor match conditions shows significantly lower saccade accuracy when saccade latency is short and high saccade accuracy when saccade latency is long (see Fig. 4). While the overall patterns look relatively similar between the methods, the SMART method again clearly shows a more reliable (less variable) temporal estimate across the different temporal resolutions (see Fig. 6a and Table 1).

- 1. Dataset 2—Saccade curvature

Dataset 2 was used to demonstrate the analysis of one-sample testing with permutation testing against baseline. The original paper showed at what intersaccadic interval saccade curvature shifts from curvature away from the predisplaced distractor location (positive curvature) to curvature away from the displaced distractor location (negative curvature). Thus, one measure is of particular interest: the estimated switch time between positive and negative curvature. This time is estimated as the center point between the offset of positive curvature and the onset of negative curvature. The offset of positive curvature was defined as the last bin/time point with significant positive curvature. The onset of negative curvature was defined as the first bin/time point with significant negative curvature. The duration between the offset and onset is considered as the precision of the estimated switch time.

Fig. 5 Results for Dataset 2. Columns 1–3: Saccade curvature as a function of the intersaccadic interval when using Vincentizing, hard-limit bins and Gaussian smoothing, respectively. The shaded gray area is the estimated number of trials per millisecond (right axis), estimated with the same kernel size as the one used for the SMART procedure. Column 4: The permutation distribution against baseline. Further details as in Fig. 4. (Color figure online)

As expected, all three methods replicate the findings by van Leeuwen and Belopolsky ([2018](#B15)): The curvature shifts from curvature away from the original location to curvature away from the displaced location (see Fig. 5). While the overall patterns look similar between the methods, the SMART method clearly shows a more reliable (less variable) temporal estimate across the different temporal resolutions (see Fig. 6b and Table 1).

Fig. 6 The temporal estimates of significant differences in Datasets 1 and 2 as a function of the number of bins (for Vincentized and hard bins) or as a function of the standard deviation (σ) of the Gaussian kernel (for SMART). **a** The estimated time when the two conditions (target match and distractor match) no longer differ from each other, from Dataset 1. **b** The estimated saccade curvature switch times, the center point between the borders of the two significant clusters in Fig. 5, from Dataset 2, with error bars reflecting the precision of this estimate (see Method section for details). (Color figure online)

Table 1 Means and standard deviations (*SD*) of the temporal estimates for each method depicted in Fig. 6 across different number of bins and values of σ

|  | Offset in Dataset 1 (ms) | | Switch in Dataset 2 (ms) | |
| --- | --- | --- | --- | --- |
|  | Mean | *SD* | Mean | *SD* |
| Vincentizing | 321 | 21 | 186 | 12 |
| Hard-limit bins | 339 | 52 | 168 | 5 |
| SMART | 329 | 19 | 179 | 4 |

The offset is the estimate of the time when the proportion correct in Fig. 4 stops being significantly different between conditions. The switch is an estimate of the moment that the curvature changes sign in Fig. 5. All values are rounded down to the nearest millisecond

1. Discussion

In the present paper, we introduced the SMART method for analyzing the time-course of response data as an alternative to the common practices of binning. The SMART method provides all-in-one solution: It reconstructs a time-series with high temporal precision and performs statistical analysis on it. The SMART method returns an event-related time-course, similar to constructing event-related potential in EEG research. By implementing a method for weighing each reconstructed data point by the amount of data contributed by each specific participant, the method is highly suitable for datasets with large variability in response-time distributions across participants. This also assures that the reconstructed time-course is continuous and without interruptions, unlike previous implementations (Maij et al., [2010](#B8); Maij et al., [2009](#B9); Maij, Brenner, & Smeets, [2011](#B10)). The SMART method takes an objective approach to the determination of cluster significance by implementing cluster-based permutation testing. To our knowledge, it is the first time that a cluster-based permutation method (Fahrenfort et al., [2017](#B3); Maris & Oostenveld, [2007](#B11)) has been adapted for one-sample-per-trial response data.

The temporal resolution when applying Vincentizing differs between bins, as bins vary in width depending on the distribution of data for each participant. It is therefore impossible to perfectly match the temporal resolution of the SMART method to Vincentizing. For hard-limit bins, the limits are set after the distribution of the temporal variable is known, and then the width of the bins can be determined by dividing the range of values by the number of bins. As this range differs, the temporal resolution for a set number of bins differs between the two datasets we used. For the hard-limit bins in the first dataset, the corresponding σs are: 66 ms, 50 ms, 40 ms, 33ms, and 28 ms, respectively, in the SMART method. For the hard-limit bins in the second dataset, the temporal resolution of 3, 4, 5, 6, and 7 bins correspond to a σ of 33 ms, 25 ms, 20 ms, 16 ms, and 14 ms. In the current paper, we choose to keep the temporal resolution for the SMART method identical for the analysis of both datasets, showing that the temporal resolution does not depend on the distribution of the data.

We systematically compared the SMART method with Vincentizing and the hard-limit binning. The SMART method has several advantages compared to binning. The width σ of the smoothing kernel has a negligible effect on the temporal estimates derived from the SMART method as indicated by an almost flat line in Fig. 6 and by the very low standard deviation across temporal resolutions (see Table 1). In contrast, the estimates resulting from both binning methods are strongly affected by the temporal resolution as indicated by the jagged line in Fig. 6 and the high standard deviation across the number of bins (see Table 1). Instead of down-sampling the available data to a few bins, the SMART method can obtain an arbitrary temporal resolution, only limited by the density of the data. Any combination of a dependent variable and a continuous independent variable (e.g., height, age, weight, speed) can be analyzed using SMART—in other words, it can be used to analyze any data which would traditionally be binned. The SMART method was not created to analyze the types of data which Vincentizing was originally designed for: participant-specific data distributions such as reaction-time distributions (Ratcliff, [1979](#B12); Vincent, [1912](#B16)). But we feel it is important to note that it is theoretically possible to adapt SMART to this type of data analysis. This can be done by changing the procedure such that the data are smoothed on equally sized new time-series (with each interpolated *t* being set separately for each participant), which are centered on the participants mean or median reaction times instead. This approach would be similar to Vincentizing and would yield a “Vincentized” temporal pattern. Considering that experimental psychology research often concerns data which *are* event related, one should be wary of using Vincentizing if there is a large variability in reaction times between participants.

However, there are some notable caveats. The SMART method does require the researcher to choose an arbitrary σ for the smoothing kernel. Although we show here that the choice of σ has a negligible effect on the reconstruction of the time-course, it does affect the frequency content and noise level of the reconstructed time-course. The smoothing procedure essentially acts as a low-pass filter. In order to avoid removing any potential high-frequency information, the smallest σ value should be used that leads to an acceptable amount of noise. The more data, the less noise, so for a given σ, noise will be largest at response times with little data.

Furthermore, it is worth noting that the SMART method can be used with any kernel of choice (not just the Gaussian kernel, although it is the most common for this type of analysis (Boon et al., [2018](#B1); Maij et al., [2010](#B8); Maij et al., [2009](#B9); van Leeuwen & Belopolsky, [2018](#B15)). Similarly, the SMART method is not limited to the sum of t-values statistics for cluster-based permutation testing or any other test statistic, if deemed more appropriate, these can be substituted (Maris & Oostenveld, [2007](#B11)).

In the present paper, we presented the SMART method—a novel approach for analyzing response data as a time-series. We provide tools for reconstructing the time-course with a higher temporal resolution compared to traditional methods of binning data. We also provide tools for performing statistical analysis on the reconstructed time-series. This powerful and flexible method can be applied to any type of one-sample-per-trial data as long as the independent variable is a continuous measure. We hope that the SMART method will become a new standard in analyzing response data.

Acknowledgements

This research was supported by an Open Research Area Grant from the Netherlands Organization for Scientific Research to Artem Belopolsky [ORA 464-15-193]. The authors are not aware of any conflicts of interest that might be perceived as affecting the objectivity of this work. We would like to thank the authors of Silvis et al. ([2015](#B14)) for the use of their data. All the code used for the analysis is available on the Open Science Framework (OSF: <https://osf.io/e5s3u/>).

Author contributions

J.v.L. and A.V.B. designed research; J.v.L. performed research; J.v.L. and A.V.B analyzed data; J.v.L., A.V.B., and J.B.J.S wrote the paper.

1. Electronic supplementary material

ESM 1 (DOCX 423 kb)

References

Boon, P. J., Zeni, S., Theeuwes, J., & Belopolsky, A. V. (2018). Rapid updating of spatial working memory across saccades. *Scientific Reports*, *8*(1), 1072. [10.1038/s41598-017-18779-9](https://doi.org/10.1038/s41598-017-18779-9)

Bullmore, E. T., Suckling, J., Overmeyer, S., Rabe-hesketh, S., Taylor, E., & Brammer, M. J. (1999). Global, voxel, and cluster tests, by theory and permutation, for a difference between two groups of structural mr images of the brain. *IEEE Transactions on Medical Imaging*, *18*(1), 32–42. [10.1109/42.750253](https://doi.org/10.1109/42.750253)

Fahrenfort, J. J., van Leeuwen, J., Olivers, C. N. L., & Hogendoorn, H. (2017). Perceptual integration without conscious access. *Proceedings of the National Academy of Sciences*, *114*(14), 3744–3749. [10.1073/pnas.1617268114](https://doi.org/10.1073/pnas.1617268114)

Gatz, D. F., & Smith, L. (1995). The standard error of a weighted mean concentration—I. Bootstrapping vs other methods. *Atmospheric Environment*, *29*(11), 1185–1193. [10.1016/1352-2310(94)00210-C](https://doi.org/10.1016/1352-2310(94)00210-C" \o "https://doi.org/10.1016/1352-2310(94)00210-C)

Godijn, R., & Theeuwes, J. (2002). Programming of endogenous and exogenous saccades: Evidence for a competitive integration model. *Journal of Experimental Psychology: Human Perception and Performance*, *28*(5), 1039–1054. [10.1037/0096-1523.28.5.1039](https://doi.org/10.1037/0096-1523.28.5.1039)

Heitz, R. P. (2014). The speed-accuracy tradeoff: History, physiology, methodology, and behavior. *Frontiers in Neuroscience*, *8*(150), 1–19. [10.3389/fnins.2014.00150](https://doi.org/10.3389/fnins.2014.00150)

Henmon, V. A. C. (1911). The relation of the time of a judgment to its accuracy. *Psychological Review*, *18*(3), 186–201. [10.1037/h0074579](https://doi.org/10.1037/h0074579)

Maij, F., Brenner, E., Li, H. O., Cornelissen, F. W., & Smeets, J. B. J. (2010). The use of the saccade target as a visual reference when localizing flashes during saccades. *Journal of Vision*, *10*(4), 7.1–7.9. [10.1167/10.4.7](https://doi.org/10.1167/10.4.7)

Maij, F., Brenner, E., & Smeets, J. B. J. (2009). Temporal information can influence spatial localization. *Journal of Neurophysiology*, *102*, 490–495. [10.1152/jn.91253.2008](https://doi.org/10.1152/jn.91253.2008).

Maij, F., Brenner, E., & Smeets, J. B. J. (2011). Peri-saccadic mislocalization is not influenced by the predictability of the saccade target location. *Vision Research*, *51*(1), 154–159. [10.1016/j.visres.2010.10.024](https://doi.org/10.1016/j.visres.2010.10.024)

Maris, E., & Oostenveld, R. (2007). Nonparametric statistical testing of EEG- and MEG-data. *Journal of Neuroscience Methods*, *164*(1), 177–190. [10.1016/j.jneumeth.2007.03.024](https://doi.org/10.1016/j.jneumeth.2007.03.024)

Ratcliff, R. (1979). Group reaction time distributions and an analysis of distribution statistics. *Psychological Bulletin*, *86*(3), 446–461. [10.1037/0033-2909.86.3.446](https://doi.org/10.1037/0033-2909.86.3.446)

Rouder, J. N., & Speckman, P. L. (2004). An evaluation of the Vincentizing method of forming group-level response time distributions. *Psychonomic Bulletin & Review*, *11*(3), 419–427. [10.3758/BF03196589](https://doi.org/10.3758/BF03196589)

Silvis, J. D., Belopolsky, A. V., Murris, J. W. I. I., & Donk, M. (2015). The effects of feature-based priming and visual working memory on oculomotor capture. *PLoS ONE*, *10*(11), 1–15. [10.1371/journal.pone.0142696](https://doi.org/10.1371/journal.pone.0142696)

van Leeuwen, J., & Belopolsky, A. V. (2018). Distractor displacements during saccades are reflected in the time-course of saccade curvature. *Scientific Reports*, *8*(1), 2469. [10.1038/s41598-018-20578-9](https://doi.org/10.1038/s41598-018-20578-9)

Vincent, S. B. (1912). The function of the vibrissae in the behavior of the white rat. *Behavior Monographs*, *1*(5), 84.
